# A quantitative analysis of the improvement provided by comprehensive annotation on CT lesion detection using deep learning

**DOI:** 10.1002/acm2.14434

**Published:** 2024-07-30

**Authors:** Jingchen Ma, Jin H. Yoon, Lin Lu, Hao Yang, Pingzhen Guo, Dawei Yang, Jing Li, Jingxian Shen, Lawrence H. Schwartz, Binsheng Zhao

**Affiliations:** ^1^ Department of Radiology Memorial Sloan Kettering Cancer Center New York New York USA; ^2^ Department of Radiology Columbia University Irving Medical Center New York New York USA; ^3^ Department of Radiology Beijing Friendship Hospital Capital Medical University Beijing China; ^4^ Medical Imaging Department Sun Yat‐Sen University Cancer Center State Key Laboratory of Oncology in South China Guangzhou China

**Keywords:** CT, deep learning, lesion detection, medical image dataset

## Abstract

**Background:**

Data collected from hospitals are usually *partially* annotated by radiologists due to time constraints. Developing and evaluating deep learning models on these data may result in over or under estimation

**Purpose:**

We aimed to quantitatively investigate how the percentage of annotated lesions in CT images will influence the performance of universal lesion detection (ULD) algorithms.

**Methods:**

We trained a multi‐view feature pyramid network with position‐aware attention (MVP‐Net) to perform ULD. Three versions of the DeepLesion dataset were created for training MVP‐Net. Original DeepLesion Dataset (OriginalDL) is the publicly available, widely studied DeepLesion dataset that includes 32 735 lesions in 4427 patients which were *partially* labeled during routine clinical practice. Enriched DeepLesion Dataset (EnrichedDL) is an enhanced dataset that features *fully* labeled at one or more time points for 4145 patients with 34 317 lesions. UnionDL is the union of the OriginalDL and EnrichedDL with 54 510 labeled lesions in 4427 patients. Each dataset was used separately to train MVP‐Net, resulting in the following models: OriginalCNN (replicating the original result), EnrichedCNN (testing the effect of increased annotation), and UnionCNN (featuring the greatest number of annotations).

**Results:**

Although the reported mean sensitivity of OriginalCNN was 84.3% using the OriginalDL testing set, the performance fell sharply when tested on the EnrichedDL testing set, yielding mean sensitivities of 56.1%, 66.0%, and 67.8% for OriginalCNN, EnrichedCNN, and UnionCNN, respectively. We also found that increasing the percentage of annotated lesions in the training set increased sensitivity, but the margin of increase in performance gradually diminished according to the power law.

**Conclusions:**

We expanded and improved the existing DeepLesion dataset by annotating additional 21 775 lesions, and we demonstrated that using fully labeled CT images avoided overestimation of MVP‐Net's performance while increasing the algorithm's sensitivity, which may have a huge impact to the future CT lesion detection research. The annotated lesions are at https://github.com/ComputationalImageAnalysisLab/DeepLesionData.

## 1 INTRODUCTION

Medical images such as computed tomography (CT) are widely used for detecting and reporting abnormal findings in the body. However, it is routine clinical practice for radiologists to not annotate every lesion that is found on CT due to various factors, including the negligible concern for small lesions and other practical constraints. Developing and evaluating deep learning (DL) models on the partially annotated dataset may result over or under estimation. This study sought to quantitatively investigate the effects of various proportions of lesion annotations in CT images on the performance of universal lesion detection (ULD) algorithms.

Progress in these ULD algorithms has not been rapid in medical imaging due to limited number of well‐annotated data. Privacy concerns and institutional barriers limit the number of patient images available for training ULD algorithms, and performing the extensive annotations by an expert necessary to appropriately label tumors is an arduous task. To address this issue, the DeepLesion dataset was initially contributed to the public domain in 2018. This foundational resource for the investigation of automated ULD^[^ ^1^ ^]^ contains CT image series from more than 4000 unique patients scanned at the National Institutes of Health's Clinical Center for a variety of indications. Each of these patients has one or more lesions in a wide variety of bodily regions, which were *partially* annotated by radiologists at one or more time points of the image series.

Many published literature on ULD to date have used the DeepLesion dataset to train, validate, and test their models.^[^ ^2^ ^]^ The original authors used DeepLesion to train and evaluate a 3D context‐enhanced region‐based convolutional neural network (CNN) for the ULD algorithm by stacking multiple 2D pre‐trained layers to extract 3D information.^[^ ^3^ ^]^ Others have used DeepLesion to demonstrate improvements by modifying the ULD algorithm.^[^ ^2^, ^4^ ^]^ More recently, a multitask universal lesion analysis network based on the Mask R‐CNN for joint lesion detection, tagging, and segmentation has been developed using DeepLesion.^[^ ^5^ ^]^ Finally, another iteration by Yan et al.^[^ ^6^ ^]^ proposed a lesion ensemble approach to mining lesion by multi‐dataset and intra‐patient lesion matching and achieved state‐of‐the‐art performance of 59.6% sensitivity at two false positives (FPs) per sub‐volume. However, there are no studies evaluating the number or percentage of partially annotated lesions needed for optimal AI training, and it is unclear how much incremental benefit there is in increasing the percentage of annotated lesions in medical images used for training the AI.

While the DeepLesion dataset represents a substantial advancement for the field, the annotation of these images was not complete. The DeepLesion dataset was formed by mining the bookmarks in the picture archiving and communication systems (PACS) of the authors' hospital. These bookmarks were created as part of routine clinical practice, in which radiologists perform annotation on selected lesions visible in images taken at selected time‐points. The reasons for these selections vary, resulting in sparse and inconsistent annotation of lesions. For most time‐points of the DeepLesion dataset, multiple lesions are present in the CT image, yet only half are labeled.

Little is known about how its incomplete lesion annotation might impact the sensitivity and specificity of an algorithm trained to recognize lesions using DeepLesion. The original DeepLesion publication noted that the accuracy of their ULD algorithm was substantially improved by increasing the annotation of the training dataset using regional labels (e.g., lung lesion, liver lesion) generated through unsupervised lesion categorization by the VNN‐16 CNN. (1). Likewise, Cai et al.^[^ ^7^ ^]^ used a lesion harvesting framework integrating automated detection with radiologist supervision to annotate additional 9805 previously unlabeled lesions. Although they estimated that this still left 40% of the lesions in the DeepLesion dataset unlabeled, they showed that using their added harvested lesions to augment the training set improved the ULDs by 7%–10%. Our preliminary investigations by radiologists also revealed that approximately half the visible lesions were annotated in the DeepLesion dataset.

Previous studies have not thoroughly evaluated the impact of partially annotated lesions on AI training and testing. Additionally, there remains ambiguity regarding the incremental benefits of augmenting the proportion of annotated lesions in medical images for AI training. This is particularly pertinent considering the significant resources and costs associated with annotating additional medical data.

In the current study, our objective was to quantitatively assess how the proportion of annotated lesions in CT images impacts the accuracy of ULD algorithms. We expanded the amount of annotations of the DeepLesion dataset by having three radiologists annotate all previously unlabeled lesions in one CT series for each patient. We contributed our enhanced dataset to the public domain and used it to train a ULD algorithm, MVP‐Net, to explore the effect of the percentage of annotated lesions on the algorithm's lesion detection performance. This study seeks to address three key questions: First, what is the accuracy of performance evaluation on datasets with incomplete annotations? Second, does an AI trained on comprehensively labeled lesions outperform one trained on partially labeled lesions, given the same sample size? Third, what is the extent of performance improvement when increasing the percentage of labeled lesions in CT images used for training ULDs?

## 2 MATERIALS AND METHODS

2.1

**FIGURE 1:**
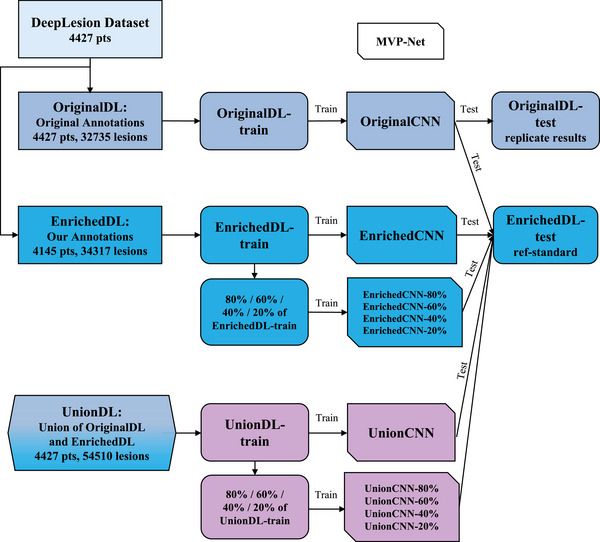
The study design. Three versions of the DeepLesion dataset were created for training MVP‐Net. OriginalDL is the publicly available, widely studied DeepLesion dataset. EnrichedDL is an enhanced dataset that features full lesion annotation. UnionDL is the union of the OriginalDL and EnrichedDL. Each dataset was used separately to train MVP‐Net, resulting in different CNN models. Then each CNN model was tested on EnrichedDL‐test set for direct comparisons. CNN, convolutional neural network; EnrichedDL, enriched DeepLesion dataset; MVP‐Net, multi‐view feature pyramid network; OriginalDL, original DeepLesion dataset; UnionDL, union of OriginalDL and EnrichedDL.

This study utilized three datasets derived from the DeepLesion dataset. The first Original DeepLesion Dataset (OriginalDL) was taken directly from the open‐source, partially labeled DeepLesion dataset. Enriched DeepLesion Dataset (EnrichedDL), the second dataset, consisted only of images from the DeepLesion series in which all lesions were fully annotated by our radiologists. The third dataset, Union of OriginalDL and EnrichedDL (UnionDL), was the union of the first two, including all images and offering the maximum annotation available for each. We used each of these datasets to train and evaluate ULD algorithms developed using a novel open‐source DL method called MVP‐Net. To show that incomplete annotation of the testing‐set causes overestimation of the AI's performance, we trained MVP‐Net on the OriginalDL training set and then compared its performance using the testing set of OriginalDL versus EnrichedDL. Next, to test the effect of higher percentage of annotations in training set, we trained MVP‐Net on the training sets of OriginalDL, EnrichedDL, and UnionDL, and then compared the performances of the resulting models on the EnrichedDL testing set. In addition, we also measured the performance of AI at incremental annotations in the training set by training the MVP‐Net on 20%, 40%, 60%, and 80% annotated lesions of EnrichedDL and UnionDL datasets to estimate the asymptote for the number of annotations needed for highest performance of lesion detection. The study design is shown in Figure 1. Our study was designed to quantitatively assess the extent to which the performance of lesion detection algorithms enhances as more annotated data is incorporated. This approach aimed to provide a quantitative understanding of the relationship between lesion annotation volume and lesion detection accuracy in CT images. OriginalCNN, EnrichedCNNs, and UnionCNNs are the same MVP‐Net architecture but trained on datasets with differing volumes of lesion annotations to investigate the relationship.

## 3 DATASETS

### 3.1 The DeepLesion dataset

DeepLesion, the National Institutes of Health's Clinical Center large‐scale, publicly available dataset, contains CT scans from 4427 unique patients and their 14 601 CT image series with 10 594 scan time‐points. Each image series covers multiple lesions. Approximately half of these have been annotated via a previously described process of harvesting PACS records, such that 32 735 lesions were annotated with a rectangular box in the central image of each lesion.^[^ ^1^ ^]^ Another automated process classified each of these marked lesions to one of the following eight sites: the abdomen, bone, kidneys, liver, lungs, mediastinum, pelvis, and soft tissue (miscellaneous lesions in the body wall, muscle, skin, fat, limbs, head, and neck). For our study, we created three versions of the DeepLesion dataset as summarized in Table 1 and described below.

**TABLE 1:** Numbers of patients, scan time‐points, image series, and annotated lesions for training, validation, and test sets of each dataset.

		#Patients	#Time‐points	#Series	#Lesions
OriginalDL	Training	3059	7386	10 224	22 919
	Validation	703	1599	2193	4889
	Test	665	1609	2184	4927
	Subtotal	4427	10 594	14 601	32 735
EnrichedDL	Training	2867	3084	4178	24 254
	Validation	659	716	970	4902
	Test	619	674	900	5161
	Subtotal	4145	4474	6048	34 317
UnionDL	Training	3059	7386	10 224	38 517
	Validation	703	1599	2193	7808
	Test	665	1609	2184	8185
	Subtotal	4427	10 594	14 601	54 510

Abbreviations: OriginalDL, original DeepLesion dataset; EnrichedDL, enriched DeepLesion dataset; UnionDL, union of OriginalDL and EnrichedDL.

### 3.2 OriginalDL

OriginalDL was formed by downloading the DeepLesion dataset from https://nihcc.app.box.com/v/DeepLesion. Training, validation, and test sets were formed for OriginalDL using the split pre‐defined by DeepLesion's random stratification at the patient level (70% training, 15% validation, 15% test) and labeled as OriginalDL‐train, OriginalDL‐val, and OriginalDL‐test, respectively.

### 3.3 EnrichedDL

EnrichedDL provides annotation of all lesions present within a subset of the patients and time points from OriginalDL.

#### 3.3.1 Lesion annotation

Three participating radiologists labeled all lesions present in the selected images with a rectangle, which was placed around each lesion in the central image of its 3D lesion volume. Our radiologists utilized the customized Weasis software^[^ ^8^ ^]^ to meticulously annotate all solid lesions found from the lung apex to the bottom of the pelvis. This included LNs, which were annotated if the short axis measured 10 mm or more. For other types of lesions, we annotated all discernible abnormalities, irrespective of their size. This comprehensive approach ensured a more complete and accurate representation of lesions within the dataset.

#### 3.3.2 Lesion classification

The radiologists assigned a location to each marked lesion according to the following 21 lesion sites, expanded from the eight classifications used in the original DeepLesion: abdomen, abdomen LN, adrenals, axillary LN, bone, inguinal LN, kidneys, liver, lungs, mediastinum LN, neck LN, ovaries, pancreas, pelvic LN, pelvis, pleura, retroperitoneal LN, soft tissue, spleen, stomach, and thyroid.

#### 3.3.3 Patient selection

To mitigate the highly time‐consuming workload of lesion annotation, patients with more than 30 lesions were excluded from EnrichedDL (*n* = 282).

#### 3.3.4 Time‐point selection

For this study, the radiologists selected the single scan time‐point for each patient in which the greatest number of lesions was present. For most patients (*n* = 4039) only this one time‐point was fully annotated. Because our goal was to create the most comprehensively annotated dataset possible within our workload limitations, we also included 106 patients from the Deep Lesion dataset for whom lesions had been annotated at all time points as part of a previous study.

#### 3.3.5 Summary

4145 patients with no more than 30 lesions per patient were included. 12 542 lesions were already annotated in these patient images. Our annotation added 21 775 lesions which had previously been unlabeled for a total of 34 317 annotated lesions. Applying the same patient‐level splits provided by DeepLesion, we created EnrichedDL‐train, EnrichedDL‐val, and EnrichedDL‐test sets.

#### 3.3.6 Proportional datasets

We created another four versions of the EnrichedDL‐train dataset which randomly selected 80%, 60%, 40%, and 20% labeled lesions for model training. These are referred to as EnrichedDL‐80%, EnrichedDL‐60%, EnrichedDL‐40%, and EnrichedDL‐20%, respectively.

### 3.4 UnionDL

UnionDL was created as the union of OriginalDL and EnrichedDL to offer a new reference set containing the most comprehensive set of patients and annotations available from our work.

#### 3.4.1 Lesion annotation

UnionDL contains annotations from EnrichedDL where available and from OriginalDL where EnrichedDL annotations were not performed.

#### 3.4.2 Lesion classification

Not performed.

#### 3.4.3 Patient selection

All 4427 patients from DeepLesion are included in UnionDL. Patients who had more than 30 lesions were not included in EnrichedDL, so all annotations for these patients came from OriginalDL.

#### 3.4.4 Time‐point selection

All 10 594 scan time‐points from DeepLesion are included in UnionDL.

#### 3.4.5 Summary

UnionDL contains 4427 patients, 10 594 scan time‐points, 14 601 image series, and 54 510 annotated lesions (32 735 from the original DeepLesion and 21 775 added through our work). Applying the same patient‐level splits provided by DeepLesion, we created UnionDL‐train, UnionDL‐val, and UnionDL‐test sets.

#### 3.4.6 Proportional datasets

We created another four versions of the UnionDL‐train dataset which randomly selected 80%, 60%, 40%, and 20% labeled lesions for model training. These are referred to as UnionDL‐80%, UnionDL‐60%, UnionDL‐40%, and UnionDL‐20%, respectively.

## 4 LESION DETECTION MODELS

### 4.1 MVP‐Net

**FIGURE 2:**
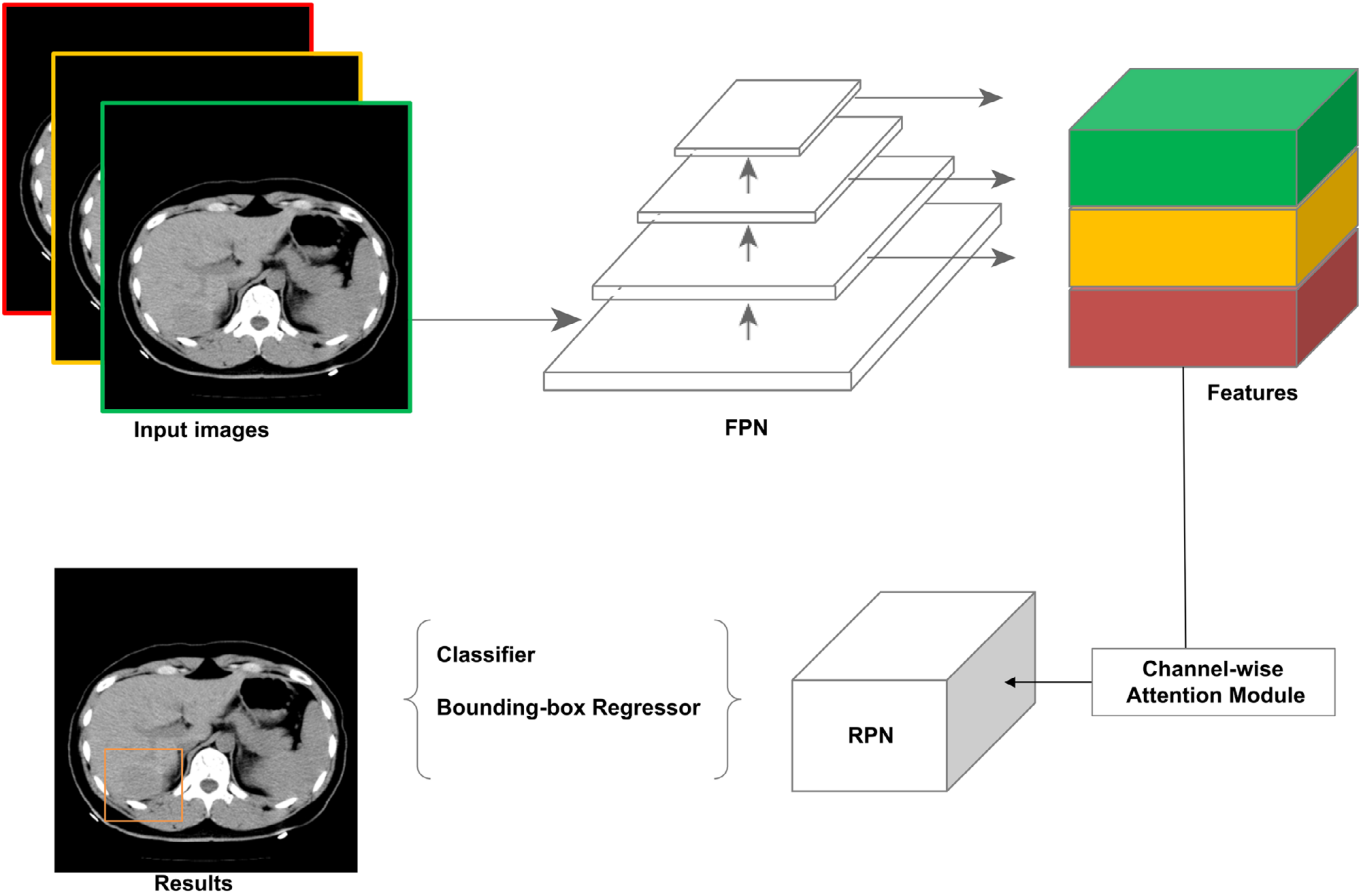
A conceptual illustration of MVP‐Net. The inputs are nine consecutive slices multiplying three CT windows to leverage the context among *z*‐directions and intensity information for different organs and lesions. FPN extracts multi‐scale and multi‐windowing features that can be properly aggregated and adaptively reweighted for different views with a channel‐wise attention module. Finally, RPN with detection headers outputs the results. CT, computed tomography; FPN, feature pyramid network; MVP‐Net, multi‐view feature pyramid network; RPN, region proposal network.

MVP‐Net is a publicly available DL method consisting of a multi‐view feature pyramid network of varied window widths and levels that applies a position‐aware attention module to effectively combine the multi‐view information.^[^ ^2^ ^]^ The inputs are nine consecutive slices adjacent to the center slice (four slices in each direction from the center slice). The outputs are the bounding‐boxes of detected lesions with corresponding confidence scores only on the center slice. A conceptual illustration is shown in Figure 2.

### 4.2 Models

MVP‐Net was trained on each of the datasets described above to create corresponding CNN models. All CNNs used the same architecture and hyper‐parameters as previously published.^[^ ^2^ ^]^ OriginalCNN was trained on OriginalDL‐train to replicate the original paper's results. EnrichedDL‐train and UnionDL‐train, and each of their proportional versions, were used to train EnrichedCNN, UnionCNN, EnrichedCNN‐P%, UnionCNN‐P%. The EnrichedCNN‐P% was the model trained on the data of EnrichedDL‐P%, and so is UnionCNN‐P% (P=80,60,40,20).

### 4.3 Performance evaluation

We tested all the models on the appropriate DL‐test dataset to evaluate their lesion detection performance using a standard metric, the free‐response receiver operating characteristic (FROC) curve. The sensitivities at different numbers of FPs per slice were calculated to show the recall at different precision levels. Mean sensitivity was the average value of the sensitivities at 0.5, 1, 2, 3, and 4 FP/image. The DeepLesion dataset originated from clinical routines where typically only the key slice of a lesion is annotated. The per‐slice metric specifically to reflect the AI's performance in accurately detecting lesions on these key slices. The lesion segmentation was not involved in this study.

## 5 RESULTS

### 5.1 Lesion size

**FIGURE 3:**
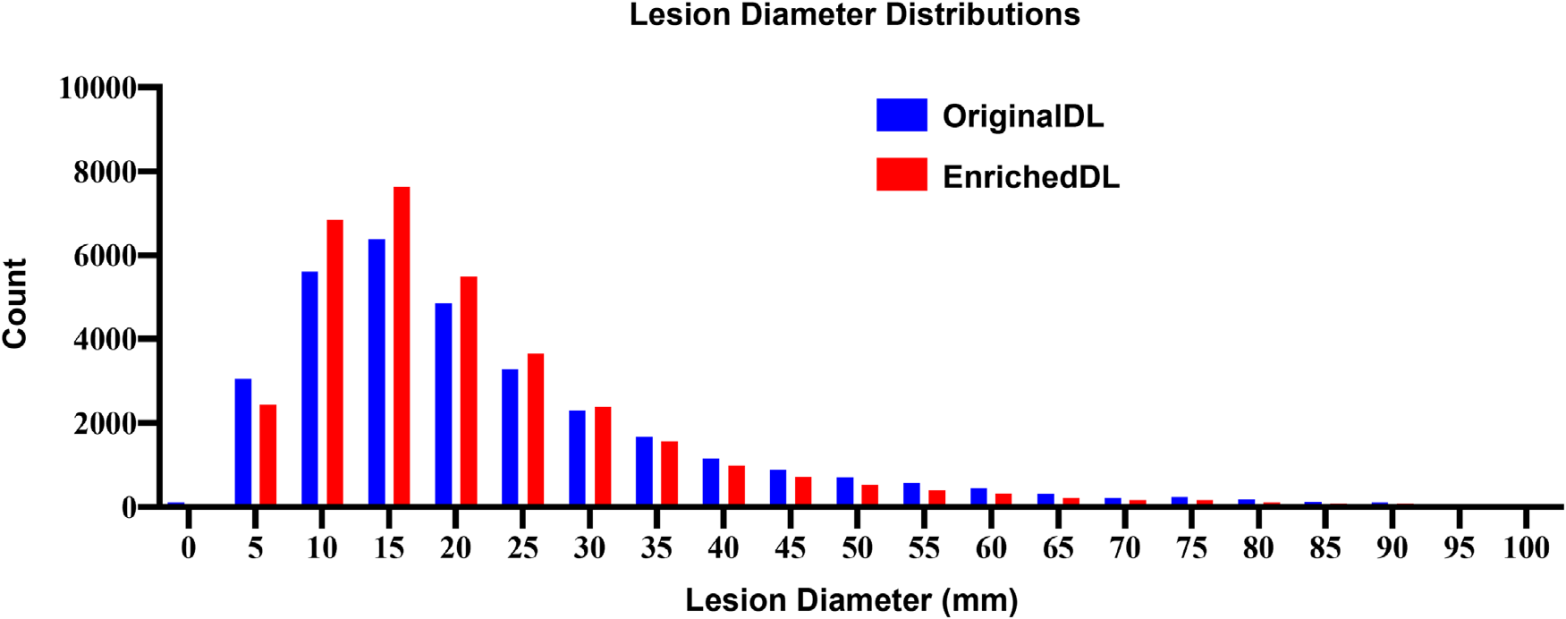
Distribution of lesion longest diameter. There were more lesions with diameters 10–30 mm in EnrichedDL compared to OriginalDL. EnrichedDL, enriched DeepLesion dataset; OriginalDL, original DeepLesion dataset.

Based on the publicly accessible DeepLesion dataset, we created a fully annotated, reference‐standard lesion dataset, EnrichedDL. The mean and standard deviation of the longest diameter (mm) of lesions in OriginalDL is 24.5 ± 20.6 while in EnrichedDL, it is 22.5 ± 17.8. The diameter distribution is shown in Figure 3. The lesion site‐wise diameter statistic is shown in Table 2. The lungs and the liver are the two most common lesion sites. Among all the lesion sites, the lesions with the smallest average size are in the lungs, and the largest are in the stomach and the pelvis.

**TABLE 2:** Lesion count and longest diameter for EnrichedDL.

Site	Count (percentage)	Longest diameter (mm) mean ± std
**Abdomen**	1721 (5.0%)	32.0 ± 26.0
**Abdomen LN**	1097 (3.2%)	22.7 ± 13.9
**Adrenal**	514 (1.5%)	25.0 ± 15.4
**Axillary LN**	1486 (4.3%)	23.0 ± 14.6
**Bone**	1052 (3.1%)	24.3 ± 16.8
**Inguinal LN**	427 (1.2%)	20.9 ± 11.9
**Kidney**	3657 (10.7%)	23.8 ± 17.0
**Liver**	4711 (13.7%)	25.0 ± 19.8
**Lungs**	8402 (24.5%)	14.9 ± 12.4
**Mediastinum LN**	3709 (10.8%)	21.0 ± 11.1
**Neck LN**	508 (1.5%)	21.2 ± 13.2
**Ovary**	77 (0.2%)	36.9 ± 20.2
**Pancreas**	926 (2.7%)	24.2 ± 22.0
**Pelvic LN**	990 (2.9%)	24.1 ± 13.6
**Pelvis**	466 (1.4%)	40.8 ± 30.2
**Pleura**	803 (2.3%)	31.9 ± 23.6
**Retroperitoneal LN**	2526 (7.4%)	24.1 ± 15.6
**Soft tissue**	814 (2.4%)	34.4 ± 26.1
**Spleen**	260 (0.8%)	21.6 ± 19.0
**Stomach**	30 (0.1%)	44.0 ± 28.5
**Thyroid**	141 (0.4%)	19.2 ± 13.4
**Total**	34 317 (100.0%)	22.5 ± 17.8

*Note*: Abdomen row includes miscellaneous lesions in the abdomen that are not in the kidneys, the liver, the pancreas, the spleen, or the stomach.

Abbreviations: EnrichedDL, enriched DeepLesion dataset; LN, lymph node.

### 5.2 Replication

OriginalCNN tested on OriginalDL‐test replicates Li et al.'s work.^[^ ^2^ ^]^ The mean sensitivity of OriginalCNN was 84.27% in our replication, slightly lower than the sensitivity of 84.82% in the original paper but within normal fluctuations in the training stage (Table 3, column 2 and 3).

**TABLE 3:** Performances of three models.

Sensitivities @ FP/image	Original paper^[^ ^2^ ^]^	OriginalCNN tested on OriginalDL‐test	OriginalCNN tested on EnrichedDL‐test	EnrichedCNN tested on EnrichedDL‐test	UnionCNN tested on EnrichedDL‐test
Mean	84.82%	84.27%	56.10%	65.98%	67.80%
0.5 FPs	73.83%	73.13%	41.63%	45.61%	47.90%
1 FP	81.82%	81.43%	52.33%	59.23%	60.52%
2 FPs	87.60%	86.91%	59.52%	70.90%	72.15%
3 FPs	89.57%	89.23%	62.59%	75.61%	77.70%
4 FPs	91.30%	90.66%	64.42%	78.53%	80.71%

*Note*: Mean is the average sensitivity of those at 0.5, 1, 2, 3, and 4 FPs/image.OriginalCNN testing on OriginalDL‐test is the replicating results of the original paper.

Abbreviations: EnrichedDL, enriched DeepLesion dataset; FP, false positive; OriginalDL, original DeepLesion dataset.

### 5.3 Effect of partial annotation of the testing set on estimation of AI performance

We examined whether the performance of a model tested on a partially labeled testing set would be estimated differently using a fully labeled testing set. OriginalCNN tested on OriginalDL‐test set achieved a mean sensitivity of 84.27% but tested on EnrichedDL‐test set yielded a mean sensitivity of 56.10%. OriginalCNN's performance on EnrichedDL‐test substantially reduced from the estimation using OriginalDL‐test (Table 3, columns 3 and 4). Partially annotated testing set missed some lesions that resulted overestimation of lesion detection performance.

### 5.4 Effect of increasing annotation of the training set

We trained MVP‐Net on the training sets of OriginalDL, EnrichedDL, and UnionDL, and compared the performance of the resulting models on the EnrichedDL testing set. OriginalCNN yielded a mean sensitivity of 56.10%, compared to 65.98% for EnrichedCNN and 67.80% for UnionCNN. The same pattern of performance, OriginalCNN < EnrichedCNN < UnionCNN, was observed in the sensitivities at each FP level (Table 3, columns 4, 5, and 6).

### 5.5 Model performances across different percentage of annotated lesions

**FIGURE 4:**
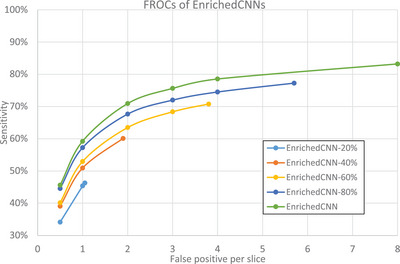
The FROC curves of MVP‐Net models trained on different proportions of EnrichedDL‐train and tested on EnrichedDL‐test. The legend shows the total number of training lesions. The sensitivities increased with the number of lesion annotations used for training. EnrichedDL, enriched DeepLesion dataset; FROC, free‐response receiver operating characteristic; MVP‐Net, multi‐view feature pyramid network.

We explored how the degree of lesion annotation in the training set can affect the performance of CNN models. We trained MVP‐Net on the EnrichedDL‐train, EnrichedDL‐80%, EnrichedDL‐60%, EnrichedDL‐40%, and EnrichedDL‐20%. Overall, the sensitivities increased when datasets with more annotated lesions were used for training as shown in Figure 4.

**FIGURE 5:**
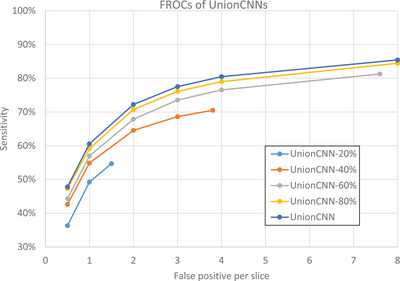
The FROC curves of MVP‐Net models trained on different proportions of UnionDL‐train and tested on EnrichedDL‐test. The legend shows the total number of training lesions. The sensitivities increased with more lesion annotations used for training, but the margin of increase in performance gradually diminished. EnrichedDL, enriched DeepLesion dataset; FROC, free‐response receiver operating characteristic; MVP‐Net, multi‐view feature pyramid network; UnionDL, union of OriginalDL and EnrichedDL.

We performed the same experiment to train MVP‐Net using the proportional UnionDL‐train sets. The result is presented in Figure 5. Again, the sensitivities increased with increased annotated lesions for the training set, but the margin of increase gradually diminished. The performance of the UnionCNN trained using 38k labeled lesions was similar to that of the UnionCNN‐80% trained using 30k labeled lesions. For example, at 2 FP/image the sensitivity of UnionCNN was 72.3% while that of UnionCNN‐80% was 70.8%.

### 5.6 Performance of fine‐grained lesion sites

Table 4 shows the detection sensitivities of different lesion sites at 2 FPs/image. Note that this method does not predict the type of each detected lesion, so the FP is the average number of FPs of all lesion types per image. The sensitivity of inguinal LN is the highest and that of bone is the lowest.

**TABLE 4:** The sensitivities on the EnrichedDL‐test of EnrichedCNN at 2 FPs/image with respect to different lesion sites.

Site	EnrichedCNN
**Abdomen**	63.50%
**Abdomen LN**	68.50%
**Adrenal**	69.80%
**Axillary LN**	84.70%
**Bone**	43.50%
**Inguinal LN**	85.70%
**Kidney**	75.80%
**Liver**	76.40%
**Lung**	73.80%
**Mediastinum LN**	76.80%
**Neck LN**	64.00%
**Ovary**	58.30%
**Pancreas**	58.30%
**Pelvic LN**	76.30%
**Pelvis**	58.70%
**Pleura**	60.30%
**Retroperitoneal LN**	70.40%
**Soft tissue**	47.30%
**Spleen**	77.10%
**Stomach**	50.00%
**Thyroid**	52.90%

Abbreviations: CNN, convolutional neural network; EnrichedDL, enriched DeepLesion dataset; FP, false positive, LN, lymph node.

## 6 DISCUSSION

The most commonly occurring types of cancer are lung cancer, prostate cancer, colorectal cancer, and gastric cancer in males, and breast cancer, colorectal cancer, lung cancer, and cervical cancer in females.^[^ ^9^ ^]^ All types of cancer involve abnormal cell growth that can invade or spread to other parts of the body causing harm and, potentially, death.^[^ ^10^ ^]^ In order for AI to be able to learn to detect all abnormal lesions in the whole body, it is crucial to collect a good quality and quantity dataset for training and testing. By expanding the amount of annotations of the largest open‐source lesion‐labeled dataset created to assist in the development of AI methods for automated detection of lesions throughout the body using CT images, we were able to identify how the partial annotation in DeepLesion limits its suitability, and provide a new reference dataset for use by other researchers to overcome these shortcomings.

Our work extended the existing body of literature by providing concrete answers to the three questions posed in the introduction. First, in response to our initial query about the accuracy of performance evaluation on datasets with incomplete annotations, our findings indicate that utilizing an incompletely‐annotated testing set tends to result in an overestimation of the AI's performance in the testing stage. When the performance of MVP‐Net was evaluated using a fully annotated testing set (EnrichedDL‐test), its mean sensitivity was 56.10%, an absolute 29% decrease from the published 84.82% performance using the original partially annotated testing set (OriginalDL‐test). The result of the original partially annotated testing set was higher(overestimated) but inaccurate because many lesions were not annotated for sensitivity evaluation. This highlights the importance of a fully annotated testing set in ensuring accurate performance assessment.

Second, addressing the question of whether AI trained on comprehensively labeled lesions outperforms one trained on partially labeled lesions with the same sample size, we demonstrated that DL models trained with partially labeled lesion annotations at multiple scan time‐points perform less well than models trained with fully labeled lesion annotations at fewer scan time‐points in the training stage. The average sensitivity of models trained on the fully labeled images was higher than that of the models trained on the similar amount of partially labeled images by an absolute value of 9.9%. This is likely because partial annotations in CT images have many unlabeled lesions which were treated as healthy tissues during training. This underscores the value of a fully annotated training set for optimizing AI efficacy.

Third, pertaining to our inquiry into the extent of performance improvement with an increased percentage of labeled lesions in CT images for ULD training, our study provides quantifiable evidence as follows.

Our results showed a gradual increase in performance as CNNs were trained on datasets with more annotated lesions. The original DeepLesion study also showed a similar trend.^[^ ^1^ ^]^ When training the model on UnionDLs, the detection sensitivity was improved as more annotations. However, the margin of increase became smaller when the training number of labeled lesions hit 30k. Due to the limited number of labeled lesions (38k) in the training set, we were not able to identify the required minimal number for labeled lesions to achieve a high and stable sensitivity. The quantitative analysis for performance improvement as a function of training data size needs further exploration in below.

**FIGURE 6:**
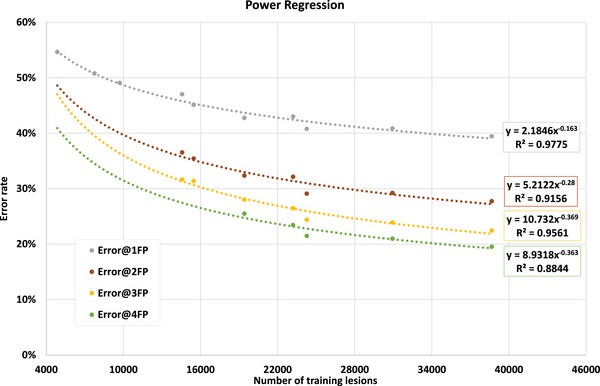
Exponential regression of training data size versus error rate of lesions class. Error rate is 1 ‐ sensitivity.

For some simple DL networks, it has been mathematically proven that solutions with generalized properties do exist, and the network's error rate was associated with the order of $x^{-0.5}$, where x is the size of the training dataset.^[^ ^11^, ^12^ ^]^ Similar work has not been performed for complex DL networks. Therefore, we did an exponential regression to calculate the order of the error rate as a function of the size of the training set as shown in Figure 6.

Based on our study results, the regression of error rate of lesions class showed $y\;=\;8.9318x^{-0.363}$, where x is the training data size and y is 1 – lesion sensitivity at 4 FPs. Based on this equation, 237k training lesions would result in missing lesion rate of 10%, and 14 million training lesions would result in missing lesion rate of 2.3%. The low error rate is also reflected by the ImageNet challenge which contains 14 million labeled images with the top five error rates at around 2% for most models.^[^ ^13^, ^14^ ^]^ However, getting such a large number of training data in medical applications is very challenging. Nevertheless, this power regression equation can be a guideline of sensitivity estimation for the future datasets.

The lesion error rates at 2 FP and 3 FP showed similar trends as that of 4 FP, but the trend line at 1 FP was flatter than the others. This is likely due to the high threshold for detecting lesions, which may result in missing many lesions and causing a high lesion error rate that does not improve with an increased number of training lesions.

Some limitations of our study were that ULD is a challenging task where the performance of the model differs when detecting different types of lesions. At 2 FPs/image, inguinal and axillary lymph node (LN) detection showed the highest performances of 85.7% and 84.7%, respectively. The reason could be the LN at armpits and groin regions having homogeneous backgrounds that make AI to detect lesions easier. Lesions in the spleen, liver, kidneys, and mediastinal and pelvic LN also showed higher performances of greater than 75%. It is possible that these lesions have larger training sets and have relatively less complex anatomy. The detection sensitivities of lesions in the bone and soft tissue were the lowest, at only 43.5% and 47.3%, respectively. This is likely because lesion sites such as bone and soft tissue have varied physical locations and relatively heterogeneous backgrounds when compared to other lesions, which can complicate the detection task. In another example, the current model can detect lung lesions with a sensitivity of 73.8% at 2 FP/image. As reported in the literature, lung nodule detection tasks on LUNA16 dataset can achieve 85%–90% of sensitivity at 2 FP/scan.^[^ ^15^, ^16^ ^]^ However, it is important to keep in mind that LUNA16 is a relatively easier task than the ULD because LUNA16 only needs to detect lesions within the lungs, which limits to scope of physical location and the types of lesions that the AI is trained upon, and the corresponding AI methods were also different. Nevertheless, we will continue to improve the training datasets and the ULD models for more accurate lesion detection that will be comparable to those of single organ models.

We made our UnionDL publicly available to extend the reference standard of DeepLesion database at https://github.com/ComputationalImageAnalysisLab/DeepLesionData. In addition, we showed that partially labeled dataset should not be used as a test dataset to evaluate AI algorithms as it overestimated the performance of the model.

## 7 CONCLUSION

Our EnrichedDL can be used as the new reference‐standard dataset for developing and accurately evaluating lesion detection methods. Given a similar amount of labeled lesions, the sensitivity of the ULD MVP‐Net trained on fully annotated lesions is found to be higher than that of the same model trained using the same amount of partially annotated lesions. The performance of lesion detection can be improved with an increased number of training lesions in the order of the power law.

## AUTHOR CONTRIBUTIONS

**Jingchen Ma**: Conceptualization; methodology; software; writing—original draft preparation. **Jin H. Yoon**: Methodology; visualization; writing—original draft preparation. **Lin Lu**: Conceptualization; investigation; validation. **Hao Yang**: Data curation; formal analysis; project administration. **Pingzhen Guo**: **Dawei Yang**: **Jing Li**: **Jingxian Shen**: Data curation; visualization. **Lawrence H. Schwartz**: **Binsheng Zhao**: Conceptualization; resources; writing—reviewing and editing.

## CONFLICT OF INTEREST STATEMENT

The authors have no relevant conflicts of interest to disclose.
